# Developmental differences across the lifespan in the use of perceptual information to guide action-based decisions

**DOI:** 10.1007/s00426-021-01476-8

**Published:** 2021-02-08

**Authors:** James Stafford, Matthew Rodger, Luis I. Gómez-Jordana, Caroline Whyatt, Cathy M. Craig

**Affiliations:** 1 School of Psychology, Queens University Belfast, David Keir Building, 18-30 Malone Road, Belfast, BT7 1NN N.Ireland UK; 2 Faculty of Human Kinetics, CIPER, University of Lisbon, Lisbon, Portugal; 3 Department of Psychology and Sport Science, University of Hertfordshire, CP Snow Building, Hatfield, UK; 4 INCISIV Ltd., Belfast, UK; 5 School of Psychology, Ulster University, Coleraine Campus, Cromore Road, Coleraine, BT52, 1SA Co. Londonderry UK

## Abstract

Perceptual information about unfolding events is important for guiding decisions about when and how to move in real-world action situations. As an exemplary case, road-crossing is a perceptual-motor task where age has been shown to be a strong predictor of risk due to errors in action-based decisions. The present study investigated age differences between three age groups (Children: 10–12 years old; Adults: 19–39 years old; Older Adults: 65 + year olds) in the use of perceptual information for selection, timing, and control of action when crossing a two-way street in an immersive, interactive virtual reality environment. Adults and children selected gaps to cross that were consistent with the use of a time-based information variable (tau), whereas older adults tuned less into the time-based variable (tau) to guide road-crossing decisions. For action initiation and control, children and adults also showed a strong ability to precisely time their entry with respect to the lead vehicle maximising the available time to cross and coordinating walking movements with the tail vehicle to ensure they were not on a collision course. In contrast, older adults delayed action initiation and showed difficulty coordinating self-movement with the approaching vehicle. This study and its results tie together age-based differences in the three components of action decision-making (selection, timing and control) within a unified framework based on perceptual information. The implications of these age-related differences in action decisions and crossing behaviours are discussed in the context of road safety.

## Introduction

We live in a dynamic world filled with moving objects. Vehicles, machines, playthings and other people are continually approaching and moving away from us. In these situations, we have multiple options and solutions available to us. Consider the task of navigating through a crowded airport or city. We must identify a gap between pedestrians and cross through or reject it and wait for another gap to appear. Additionally, when a gap is selected we can choose to walk, skip, or run through it. Thus, action-decisions are composed of three related processes: *selection, timing and control*.

Choosing which action is most appropriate requires the successful perception of what the environment currently affords, i.e. the spatiotemporal demands. For instance, a moving gap between pedestrians may afford the possibility for passing through at one moment and collapse into an impenetrable barrier at the next moment (Fajen, Riley, & Turvey, 2009). Furthermore, as humans and other animals have limited forces available to make adaptations to their movements, action-based decisions must be made ahead of time. Thus, successfully avoiding approaching obstacles requires successfully coupling perception and action. We require our motor systems to produce evasive actions when necessary and we require perceptual systems to prospectively control these movements. Within this mutual relationship, information is embedded that can be used to guide decisions about when and how to act. As an actor moves through the environment, the information specifying their relative position with respect to other objects will change. It is the way this information changes over time that specifies an action-relevant property of the environment to an actor, known as an “affordance” (Gibson, 1979). Being able to perceive an affordance will therefore allow the actor to determine which actions are possible and which are not as an event unfolds (Turvey, 1992). Indeed, Craig and Watson (2011) note that affordances capture the essence of what action-decisions are: knowing when certain environmental conditions afford a certain action, and the ability to prospectively guide those actions.

### Affordance perception across the lifespan

Since affordances are determined by the fit between the properties of the environment and the action capabilities of the actor, the same environmental characteristics can offer different opportunities at different stages of lifespan (Turvey, 1992). For example, the affordance for passing through a narrow doorway may be possible for a small child but impossible for an adult. Therefore, it is imperative that actors are adaptively sensitive to informational invariants that specify the Environment-Actor System (EAS) throughout their lifespan. During young adulthood when our motoric and perceptual abilities have fully developed, we appear to be sensitive to these perceptual invariants by continually performing appropriate judgements of whether a property of the environment determines the possibility of an action, such as when walking through passageways (Franchak, Celano, & Adolph, 2012; Warren & Whang, 1987), stepping across an obstacle (Cornus, Montagne, & Laurent, 1999), standing upright on inclined surfaces (Hajnal, Wagman, Doyon, & Clark, 2016), and fitting the hand into an aperture (Ishak, Adolph, & Lin, 2008). However, the time scale of when affordance perception is optimally accurate is an open-question. Indeed, laboratory studies of children’s affordance perception suggest early deficits. For example, younger children (3–5 years) make large errors when choosing to whether to reach through openings of various sizes but by 7 years children’s perception of this affordance reaches adult-like levels (Ishak, Franchak, & Adolph, 2014). In contrast, research involving making action-based decisions in relation to other fast-moving objects (e.g. vehicles) in the environment has shown that affordance perception continues to undergo change even in late childhood and early adolescence (i.e. 10–12 years; Chihak et al., 2010; Plumert & Kearney, 2018).

Similarly, there is conflicting evidence about the effects of healthy ageing on affordance perception (for a review, see; Comalli, Franchak, Char, & Adolph, 2013). In some cases, older adults (65 + years) appear make action-based decisions just as well as younger adults. For example, like younger adults, older adults can accurately judge the largest possible riser height for stair-climbing, accounting for their reduced hip flexibility (on average) and the fact they could not lift their legs as high as their younger counterparts (Konczak et al., 1992). Older adults appear to achieve this feat using a perceptual invariant which specifies the optimal ratio between stair riser height and distance taken from the stair, an environmental property determining climb-ability, in terms of the actor’s own stepping ability (Cesari, Formenti, & Olivato, 2003). However, in other tasks, older adults appear to be less accurate in their judgements of what the environment affords them. When judging the affordance of pass-through-ability between two moving vehicles, older adults often underestimate the amount of time needed to pass through safely compared to young adults (Stafford, Whyatt, & Craig, 2019). This suggests that under certain environmental constraints, children and older adults are less attuned to the relevant perceptual-based information that guides decisions about when and how to act.

### Road-crossing as a model task for dynamic affordance perception

To understand why picking up perceptual invariants appears to be particularly difficult for children and older adults in some tasks (e.g. crossing the road) and not others (e.g. stair-climbing) requires an understanding of how perceiving and actualizing affordances differ when the environment is static verses when the environment is dynamic. In static environments, actions must be fitted spatially, e.g. ‘is this passageway wide enough for me to walk through?’ Such an affordance is perceived in body-scaled terms, such as eye-height, which can be optically specified. To date, much of what we know about children and older adult’s sensitivity to specifying information involves this body-scaled, spatial information (Comalli et al., 2013; Dos Santos, Costa, Batistela, & Moraes, 2020; Finkel, Schmidt, Scheib, & Randerat, 2019; Franchak, Celano & Adolph, 2012). However, in such cases, the opportunity for action does not change over time. The size and location of a passageway remain the same regardless of when perceivers act on their decision. In contrast, decisions in dynamic environments must allow adequate time to carry out actions. A moving object may afford a possibility for action at one point in time but not at a later point in time. This means that actions must be fitted to decisions both spatially and temporally, which is not the case when perceiving and acting on affordances in a static environment. Such transient opportunities for action are prominent in traffic environments with gaps between vehicles continually appearing and dissipating. This has led to Plumert and Kearney (2018) proposing road-crossing as a model system for studying dynamic affordance perception. This is because road-crossing is composed of action decision-making’s three components: selecting a sufficiently large gap (action selection), entering the gap behind the lead vehicle but sufficiently ahead of the tail vehicle (action initiation), and reaching the opposite sidewalk before the tail vehicle reaches their line of crossing (action control).

### Ongoing development of pedestrian road-crossing skills

Since road-crossing can act as a model system for dynamic affordance perception, past research with child and older pedestrians can shed light on how these components of action decision-making change across the lifespan. For example, despite the fact that both the speed and the distance of a car affect one’s ability to safely navigate through inter-vehicle gaps, pedestrians aged 5–12 years seem to rely more on vehicle distance than speed during gap selection (Connelly, Conaglen, Parsonson, & Isler, 1998). Furthermore, after selecting what action to perform, children have been shown to delay their crossing longer than adults for similar sized gaps, increasing the risk of injury when a gap is eventually accepted (Plumert, Kearney, & Cremer, 2004; Schwebel, Davis, & O’Neal, 2012). In an attempt to understand how the linking of action selection and action initiation changes throughout development, O’Neal et al. (2018) compared children aged 6, 8, 10, 12 and 14 years old to adults in their ability to select an inter-vehicle gap from a single lane of continuous traffic and tightly time their entry into the roadway. The authors noted that both gap selection and timing improved steadily with development, reaching adult-like levels by the age 14. Interestingly, 12-year-olds appeared to compensate for their poorer timing of entry by selecting more conservative gaps than those of 6-, 8-, 10-, 14-year-olds and adults suggesting an improved calibration to their still developing action capabilities. Finally, recent research has studied the ability to coordinate self and object movement, independent of movement initiation and gap selection (Chihak, Grechkin, Kearney, Cremer, & Plumert, 2014; Chihak et al., 2010). For instance, Chihak et al. (2010) investigated how well 10- and 12-year-old children (and adults) adjust their motions to intercept (without stopping) a moving gap between two red blocks in a bicycling simulator. Block arrival times were manipulated such that participants needed to speed up or slow down to intercept the gap. The children in this study exhibited significantly more variability in their approach to the intersection and in the amount of time they had to spare. Also, as in previous road-crossing studies, the authors found the younger age groups timed their entry relative to the lead block in the gap less tightly than adults.

While this research on component skills has yielded valuable information, the picture regarding the development of children’s action decision-making is far from complete. Cisek (2007) proposed that during overt performance of movements the processes of action selection and action specification operate simultaneously and continuously and therefore should be regarded as one and the same dynamic process. As a result, more research that investigates how the three different components of action decision-making are linked (particularly how action control leads on from selection and initiation) during development is of paramount importance.

### The effects of healthy ageing on road-crossing skills

Interestingly, at the opposite end of the lifespan, older adults (65 + years) have also exhibited difficulties perceiving and actualizing affordances in traffic environments (for a review see; Tournier, Dommes, & Cavallo, 2016). As our action capabilities change with age, a greater importance is placed on the visual system’s ability to accurately detect action-relevant information with inaccurate judgements potentially having more serious health implications. Indeed, when choosing a safe gap, two studies comparing the street-crossing decisions of different age groups revealed that older adults (70–80 years old) do attempt to accommodate for a slower walking speed by choosing a larger median time gap than their younger counterparts (20–30 and 60–70 years old; Lobjois & Cavallo, 2007, 2009). However, similar to children below the age of 12, several virtual reality studies have shown that older adults tend to use simplified heuristics based on vehicle distance, which often result in deciding to cross when vehicles are moving at high speeds (Dommes & Cavallo, 2011; Oxley, Ihsen, Fildes, Charlton, & Day, 2005).

Parallels between older adults and children can also be found during action initiation. Observations of real-life crossing behaviours (Oxley et al., 1997) as well as indoor experiments (Holland & Hill, 2010) have revealed that older pedestrians take about 1 s longer than younger adults do to begin crossing. Crucially, this time spent judging the crossability of a gap takes time away from time available to successfully navigate through it. However, unlike children and adults, older pedestrians have been shown to be unable to compensate for this delay and maintain an adequate safety margin by increasing their walking speed whenever the time gap decreases (Lobjois & Cavallo, 2009; Morrongiello, Corbett, Milanovic, Pyne, & Vierich, 2015). Lobjois and Cavallo (2009) suggested that the crossing decisions and actions of younger pedestrians are much more finely tuned to time gaps because they use visual feedback while crossing to null the difference between their current walking speed and the ideal walking speed (see Fajen, 2007). In contrast, older adults may have trouble continually regulating their movements based on specifying optical information. No previous work has directly assessed lifespan changes in how crossing decisions and ongoing actions in traffic environments are regulated by visual information.

### Optical specification of gap-closure information

Together, these studies of child and older pedestrians suggest that ability to link the three different components of action decision-making is undergoing developmental change at least into late childhood and is prone to age-related change later in life. To overcome the problem of what to do and how to do it, gap decisions and crossing actions must be coupled together by information that can be used to guide both the choice of behaviour and continuous fine-tuning of actions. This would require a single source of information that specifies properties of the environment-actor system and is suitable for the continual guidance of action (Stoffregen, 2000). Time-to-arrival (TTA) has been identified as such an action-relevant informational quantity which has been shown to be used for deciding *when* to initiate a crossing, as well as to determine whether one needs to *adjust* the speed of movement during the crossing to avoid collision (Morrongiello et al., 2015). David Lee’s (1976) seminal paper provided a formal description of perceptual information directly available from the environment which can directly specify the TTA of an approaching object. Tau, describes the time to closure of a motion gap at its current closure rate and is defined mathematically as the size of the gap, at any given moment, divided by its rate of closure ($x$/$\dot{x}$) (Lee, 1998). Optically, tau can be picked up from the expanding image of an approaching object projected on the retina. Importantly, Lee (1998) highlights that tau enables the prospective selection and guidance of action, as it provides information about the way the gap is closing that can be extrapolated into the future to adjust and control goal-directed movement.

### Crossing two lanes of traffic

Judging the TTA of multiple lanes of traffic is particularly challenging because the gaps (which cannot be viewed simultaneously) approach from opposite directions. When faced with multiple lanes of traffic, two types of scenarios can emerge. When the far gap opens before or with the near gap, the temporal window for action appears as a single “aligned” gap spanning both lanes of traffic. Conversely, in a “rolling” gap, the near-lane gap opens before the far-lane gap allowing pedestrians to begin crossing (if the temporal offset is sufficiently large) before the far-lane gap has opened. To investigate pedestrian’s gap preferences when faced with multiple lanes of traffic, Grechkin, Chihak, Cremer, Kearney, and Plumert (2013) monitored 12- and 14-year-olds and adult’s gap selection while crossing through a series of 12 intersections in a bicycling simulator. All age groups exhibited a preference for rolling over aligned gaps, accepting more gaps and having more time to spare, despite arguably being a more difficult task. Presumably, this was because the pedestrians recognized the potential for rolling gaps to stretch the total time available for crossing. Indeed, rolling gaps enabled a 49% gain in time-to-spare in the far lane and a significant 8.7% gain in the mean time remaining until the tail vehicle closed the gap. In particular, children had significantly less time to spare than adults when they crossed an aligned pair. Similarly, older adults show specific difficulties when crossing two-lane traffic by crossing more slowly, adopting smaller safety margins, and making more decisions which led to collisions than adults (Dommes et al., 2014, 2015).

The fact that children and older adults often prioritize safety over simplicity suggests an age-related difficulty in perceiving an affordance nested in multiple transient gaps. According to tau theory, when judging simultaneously available gaps the pedestrian must attune to the difference between the tau of each gap which will equal the duration of the up-coming spatio-temporal window that is available for crossing. If the projected time to cross is less than this spatio-temporal window then safe crossing is afforded (Lee, Young, & McLaughlin, 1984). To actualize these affordances, Grechkin et al. (2013) suggest a pedestrian could initially coarse-code a pair of near and far-lane gaps as “crossable” without mentally tracking their precise locations. If the tau of the gaps in both lanes are deemed acceptable, the pedestrian can then check back and forth to see if the overlap between the gaps is still deemed to be sufficient for safe crossing. This act of checking and rechecking would then reduce the demand for cognitive processes that require an estimation of an out-of-view object’s TTA (cf. Tresilian, 1995).

### Aim and research questions

The present study intended to identify the way a particular informational variable, such as *tau* (a time-based variable), might be used to guide decisions about what, when and how to act in traffic environments with multiple aligned lanes of traffic. As children and older adults have been identified as having difficulties across the different components of road-crossing, the question we asked in this experiment is whether these age groups differ in their ability to use task-relevant information that underpins action selection, action timing, and action control.

## Methods

### Participants

A total of 45 participants were recruited through local schools, local fitness classes and personal contacts. Fifteen children (5 boys, 10 girls) ranging in age between 10 and 12 (*M* = 11 years, SD = 0.85), 15 adults (8 men, 7 women) ranging in age between 18 and 39 (*M* = 23.5 years, SD = 4.1), and 15 older adults (5 men, 10 women) ranging in age 65–91 (*M* = 73.5, SD = 8.9) were recruited to participate in the study. All participants were able to detect the oncoming vehicle at its farthest position. The present study received ethical approval from Queens University Belfast ethics committee with parents of child participants giving informed consent and children giving informed assent.

### Experimental setup

**Fig. 1:**
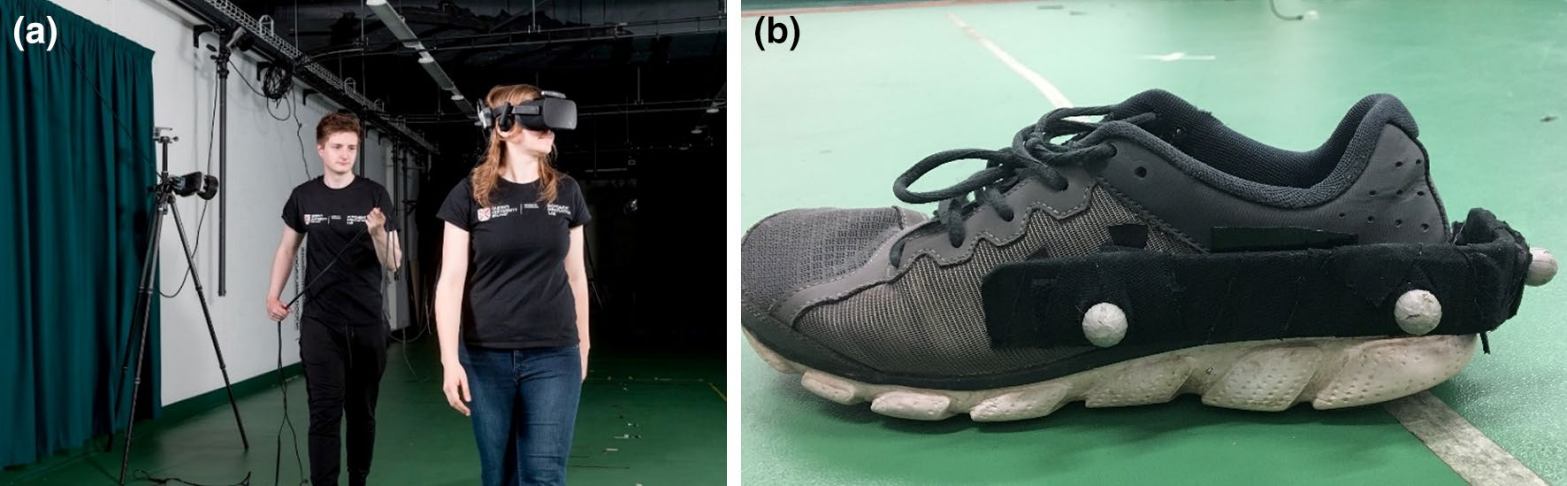
**a** Photograph (left) of a participant walking during a trial wearing the head-mounted display (HMD) with the Intersense IS-900 bar placed on top of the headset. An experimenter followed behind the participant carrying the cables to ensure safety. **b** One of the two rigid bodies attached to each foot to allow the participant to see a representation of their feet in the virtual environment

A virtual street consisting of a 6-m-wide road was presented using an Oculus Rift DK2 stereoscopic head-mounted display (see Fig. 1a). The Oculus allowed the virtual presentation to have a resolution of 1920 × 1080 refreshing at 30 frames per second. Importantly, this head-mounted display preserved an egocentric viewpoint, which is important for accurately perceiving the optic flow used when acting in real life (Craig, 2013). The visual scene consisted of a flow of oncoming traffic (18 vehicles of bi-directional traffic) with the direction of traffic following UK traffic rules (near side approaching to the right of the pedestrian). Embedded in this flow of traffic was a distinct gap in which participants had to decide whether to cross through. Vehicle speed (32, 48, or 64 km/h) and distance (30, 40, 50, 60, 70, or 80 m) were varied to give 18 unique time gaps (see Table 1). The time gaps between cars were chosen to reflect the average walking speed of an adult (1.42 m/s; Mohler, Thompson, Creem-Regehr, Pick, & Warren, 2007) which would result in 50% of the gaps/trials affording comfortable crossing (i.e. time to closure of the gap is greater than 4.2 s) and 50% not (i.e. time to closure of the gap is less than 4.2 s). Each lane of traffic was symmetrically aligned (i.e. cars in the near and far lane will arrive at the pedestrian’s line of travel at the same time). This ensured that only one “aligned” spatial–temporal gap was presented to the participant in each trial.

**Table 1:** Table displaying the 18 unique combinations of speed, distance, and the resulting time-to-arrival

Speed (KPH)	Distance (M)	Time-to-arrival (speed/distance)
32	30	1.69
48	30	2.24
32	40	2.25
32	50	2.81
48	40	2.96
64	30	3.36
32	60	3.37
48	50	3.73
32	70	3.93
64	40	4.47
48	60	4.48
32	80	4.49
48	70	5.22
64	50	5.59
48	80	5.97
64	60	6.71
64	70	7.83
64	80	8.95

**Fig. 2:**
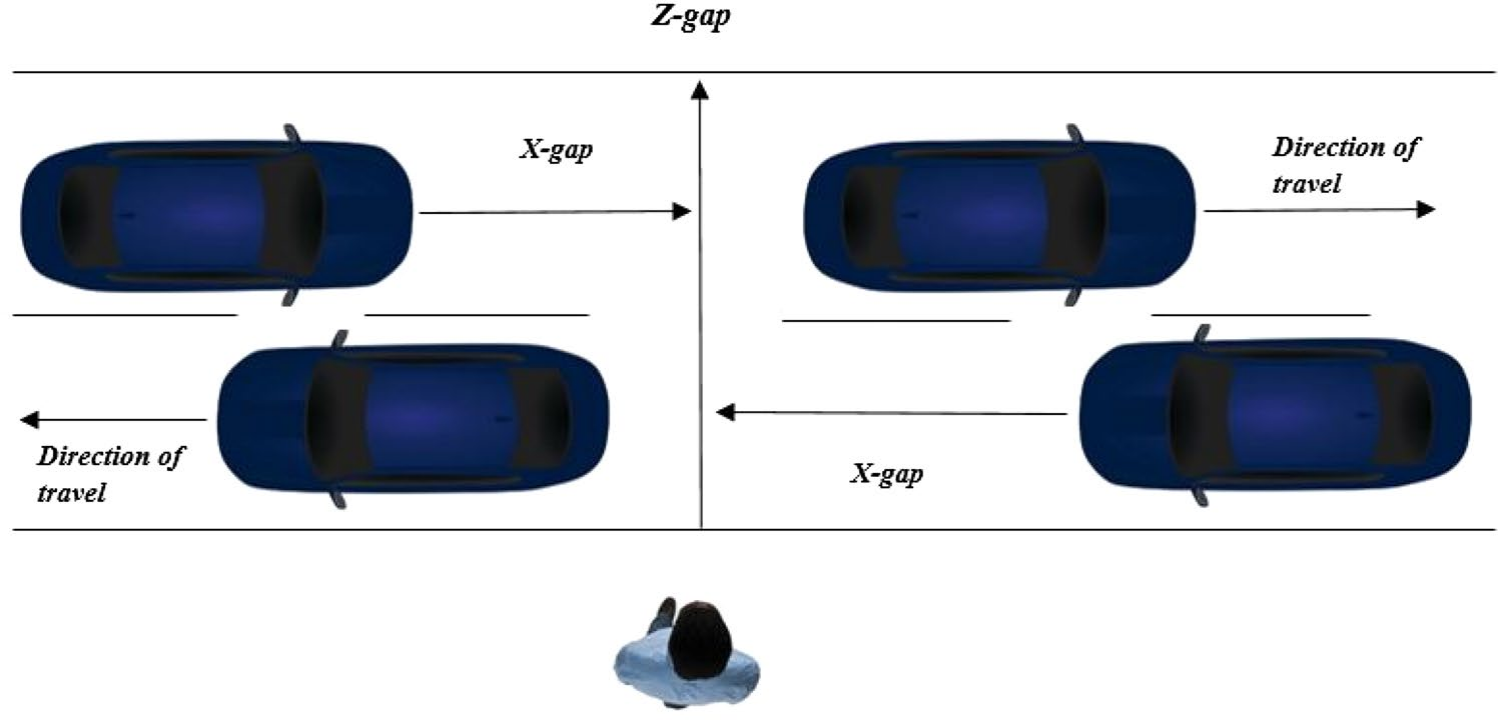
A schematic diagram showing the axes of movement of the two lanes of cars and the gaps between them. The initial tau is represented by the X gap between the participant and the second vehicle at the moment the rear bumper of the first vehicle passes the participant

The participant was placed on the edge of the sidewalk facing the virtual road (see Fig. 2). The HMD had a diagonal field of view of 90 degrees and the pedestrian could look either direction by rotating their head. To ensure movement was updated in real-time while navigating through the virtual environment, the ultrasonic Intersense IS-900 Motion tracking system was used to track the participants head movement and orientation at 120 Hz. In addition to the tracking of the head, participants were able to visualise the position of their feet (in the form of two white cuboid shaped boxes). This was created using a rigid body made up of three reflective markers attached to each foot and which was detected using a set of 12 Qualisys infrared motion capture cameras (Qualisys Ltd., Göteborg, Sweden) recording at 100 Hz (see Fig. 1b). This allowed pedestrians’ real-time feedback of their movements to increase the level of presence and also help with familiarisation of walking when immersed in a virtual environment.

### Procedure

For each trial, participants were instructed to judge whether a gap within the flow of traffic would allow them enough time to safely walk across the virtual street, and to walk across the virtual street when they perceived the gap to be safe to do so. Participants were instructed to choose gaps in which they could cross the whole street at a comfortable speed and if they felt they needed to adjust their speed during the trial, this was permitted. Participants were further instructed that if they felt the gap between cars was too small, they should just wait until the trial was over and see if the gap between cars in the next trial afforded crossing.

Each session began with the experimenter explaining what the participant should do. Participants were then asked to put on the HMD and look around the virtual environment to explore their surroundings. Two experimenters were always present when the experiment was carried out. One walked alongside the participant holding the cable that connects the headset to the computer to ensure participant safety, while the other controlled the motion capture and virtual reality computer equipment. Once the participant felt comfortable wearing the HMD, a familiarisation period followed. This allowed participants to report if the headset was sitting comfortably, along with adjusting the inter-ocular distance in the headset to avoid blurry vision. Participants were then instructed to cross the road five times with no traffic present to gain experience walking through the virtual environment. At the start of each trial, participants returned to the same side of the crosswalk at the same point, directly facing the opposite curb. Following familiarisation, the participant performed 13 practice trials in which the order of different rates of gap-closure presentations was randomised and was not included in the main analysis. These gaps were always synchronised and thus simultaneously available in both lanes. Following this practice session, participants completed 54 pseudo-randomly presented trials with 3 repetitions of each time gap. The experiment took roughly 45 min to complete.

### Measures and data analysis

The positions (i.e. the 3-dimensional *x*, *y*, and *z* coordinates in the experimental set-up) of the participant were recorded every frame and used to calculate the following: action selection (gap acceptance, % of collisions, information for gap selection), action timing (timing of entry and variability of timing), and action control (crossing time, duration on collision course, magnitude of safety, and time to spare) measures for each participant for each trial.

#### Gap acceptance

Gaps between cars were recorded as accepted crosses when participants walked more than 1 m across the 6 m road. If the participant moved less than 1 m, gaps were classed as rejected crosses. To examine whether gap acceptance thresholds differed significantly across age groups and gap size, and whether gap sensitivity was moderated by age, a mixed-effects logistic regression was conducted. The model included fixed-effects predictors of age (as a categorical variable) and gap size (as a continuous variable), which were tested for both main and interaction effects. Likelihood ratio tests indicated that a random effect for participant should also be included in the model. In addition to reporting z-statistics and p-values, odds ratios and 95% odds ratio confidence intervals (CI) are also reported.

#### Collisions

A collision was recorded if the inter-vehicle gap closed before the participant reached the opposite sidewalk.

#### The use of prospective information for gap selection

Tau theory is based on the concept of the rate of closure of motion gaps. A tau value (or ‘time-to-contact’) is calculated by taking the current size of a gap divided by its rate of closure, mathematically represented in Eq. (1) as:

1 $${\tau }_{X}=x/\dot{x,}$$

where $x$ is the magnitude of the gap and $\dot{x}$ is its first-order derivative with respect to time. Tau is negative and approaches zero as the gap closes. The various combinations of speeds and distances in the road-crossing simulator provide a continuous scale of 18 unique initial tau values. Initial tau value is defined as the tau value (or time-to-arrival) of the second vehicle at the point the rear bumper of the first vehicle passes the participant (see Fig. 2). The closer the tau value is to zero, the less time the pedestrian has to cross safely.

By plotting the initial tau value of the inter-vehicle gap as soon as the lead car passes against the response data (% judged as pass), we are able to see if participant’s crossing behaviour is consistent with this information. Ideally, assuming participants are sensitive to the prospective informational quantity tau, an ‘S-shaped’ curve should emerge as it is expected that when the tau value is large, the % judged as crossable will be 100% and when the tau value is small, the % judged as crossable will be 0%. Onto each age group, the best possible logistic function was fitted using the Eq. (2):

2 $$y=\frac{1}{1+{e}^{-k(c-t)}}.$$

When the tau value is close to the participant’s critical value, the judgements should be more difficult and so, the % judged as crossable should reflect this and be around 50%. Next, we will investigate if the ability to prospectively judge the affordance of passability in dynamic, traffic environments based on this time-to-contact variable is different across the various age groups.

#### Timing of entry

The time (in seconds) between the tail of the lead car of the gap passing the participant and the moment the pedestrian entered the path of traffic was recorded. We also created a variability of timing score for each participant by calculating the standard deviation of the timing of entry across the 54 trials.

#### Crossing duration

The time (in seconds) between the participants entering the inter-vehicle gap to reaching the opposite sidewalk.

#### Duration of trial spent on a safe course of action

To assess if participants were tuning into the optical variable tau to adjust their walking behaviour while crossing the road, the current experiment modelled the scenario as the simultaneous closure of two gaps: one gap closes between the approaching vehicle the pedestrian (tauX) and another gap closes between the pedestrian and the opposite sidewalk (tauZ). For the participant to optically determine whether they are on a collision course and adjust their movement speed accordingly, they must be sensitive to the difference between these two tau values. Subtracting tauZ from tauX provides a continuous tau differential value which specifies the pedestrian’s ‘current future’ should both gaps continue to close at their current velocities allowing for the participant to optically determine the affordance of a dynamic gap at its current rate of closure (Watson et al., 2011). For instance.

If the value of the tau of gap *Z* (between the participant and the opposite sidewalk) is closer to zero than the value of the tau of gap *X* (between the tail vehicle and the participant), i.e. |*τ*(*Z*)| <|*τ*(*X*)| then the participant’s current walking speed is sufficient and the gap affords safe passage. This is due to the ‘current future’ dictating that the participant-sidewalk gap will close before the inter-vehicle gap.

If, however, the value of tau of gap *X* (cars and the participant) is closer to zero than the value of the tau of gap *Z* (participant and the opposite sidewalk), i.e.|*τ*(*X*)| <|*τ*(*Z*)|, then the participant’s current walking speed is not sufficient and the inter-vehicle gap affords collision. This is due to the ‘current future’ dictating that the inter-vehicle gap will close before the participant-sidewalk gap.

To assess how long a participant spent on a safe course of passage, the percentage of the cross duration in which tau value of gap *Z* is closer to zero than the tau value of gap *X* was calculated.

#### Magnitude of safety

To assess the temporal margin of safety once a participant was no longer on a collision course (i.e. when the value of the tau of gap *Z* is closer to zero than the value of the tau of gap *X*), the (normalised) area under curve of the positive part of the tau difference was calculated. The inputs were tauZ and tauX (in that order), and the output is the area under curve of the positive values only, having first interpolated the trial data into 100 samples. A larger area under curve, calculated using the Trapezoidal Integration Method, represented a greater aperture between the two taus; hence, the participant achieved a safer time gap between themselves and the approaching car during the cross. Trapezoidal Integration Method involves dividing the total area under curve into trapezoids (1 trapezoid per sample for a total of 100 trapezoids). Next, the total area under curve is calculated by adding up the area of each of the 100 trapezoids. This allowed an investigation into whether different age groups were maintaining the affordance of safe passage in different ways. For instance, children may delay the initiation of crossing longer than adults but compensate for this by accelerating towards the end of the crossing phase. As a result, even though they may still exit the gaps safely, children may have a smaller area under curve due to spending a less of the overall duration of the crossing phase with a ‘safe’ difference between the car tau and their own. Such a strategy would reflect weaker synchronization of self and object movement throughout the duration of the cross and thus, less refined action control.

#### Time to spare

The time (in seconds) until the vehicle reached the participant’s line of crossing at the moment the participant reached the opposite sidewalk.

Data were filtered using a dual-pass 5 Hz Butterworth filter in MATLAB**®**. These measures allowed us to investigate how age impacts the ability to link decisions and actions in dynamic scenarios where the temporal window for action is ever-changing.

## Results

### Analysis strategy

The main goals of the present experiment were to determine first if age is a significant constraint on perceiving and acting upon dynamic affordances and if so, are there age-related differences in the information used to specify these affordances. To investigate how the use of such an informational variable differs between people of different age ranges, crossing behaviour was organized into three main sections: (1) action selection, (2) action timing, and (3) action control. Action selection was investigated via gap acceptance, % of collisions, and how consistent gap selection was with the informational variable tau that specifies the rate of closure of the gap. Action timing was assessed using movement timing variables of timing of entry and variability of timing. Finally, action control was assessed by comparing age group’s road-crossing duration, ability to control the closure of gaps and avoid being on a collision course, and the use of tau information to maintain a temporal safety margin. These measures reflect a sequential investigation of how three age groups differed in terms of the perception of affordances, followed by how the age groups differed in their actualisation of chosen affordances.

### Action selection

#### Gap acceptance

Mixed-effects logistic regression analyses revealed that all age groups were more likely to accept larger than smaller gaps, *z* = 23.68, *p* < 0.001. These results are similar to past work indicating that features of traffic (i.e. the size of the gaps available for crossing) influence crossing decisions. In addition, gap acceptance thresholds varied with age group. Relative to adults, older adult’s gap acceptance thresholds were more conservative as this age group as they were 0.58, 95% CI [0.27, 1.18], times less likely to accept a gap of any given size. However, this effect failed to reach significance (*z* =  − 1.52, *p* = 0.13). In contrast, children were 1.23, 95% CI [0.59, 2.59], times more likely to accept a gap of any given size than adults but again, no significant differences were found between the groups (*z* = 0.55, *p* = 0.58). Compared to children, however, older adults were significantly more conservative (*z* =  − 2.07, *p* = 0.04) with the odds of accepting a gap of any size being 0.45, 95% CI [0.22, 0.96], times smaller than the odds of a child accepting a gap of any size.

To test if age group significantly moderated gap size selection, an interaction term was added to the model. Relative to adults, older adults were less discriminating in their gap choices, taking more of the smaller gaps and fewer of the larger gaps (*z* =  − 4.49, *p* =  < 0.001). No significant differences were found between children and adults (*z* =  − 1.77, *p* = 0.08). With a unit increase in gap size, children and older adults were 0.77, 95% CI [0.58, 1.0], and 0.55, 95% CI [0.42, 0.71], times (respectively) less likely to accept a gap. Similarly, compared to children, older adults were significantly less discriminating (*z* =  − 2.87, *p* = 0.004) being 0.71, 95% CI [0.56, 0.90] times less likely to accept a gap following a unit increase in gap size.

### Collisions

The proportion of crosses that resulted in collisions was significantly different across age groups *F*(2,44) = 6.601, *p* = 0.003, *η*^2^ = 0.11) indicating that age significantly impacts the ability to safely navigate traffic environments. Adults recorded the lowest proportion of crosses resulting in collisions (*M* = 1.46%, SD = 2.82, range 0–29.41%) with 11 participants recording at least one collision, children recording the second most collisions, (7.23%, SD = 7.75, range 0–9.52%) with 4 participants recording at least one collision and older adults recording the highest proportion of collisions (*M* = 15.99%, SD = 17.18, range 0–50%) with 12 (participants) recording at least one collision. A post hoc Tukey HSD test indicated there was a significant difference between adults and older adults in the number of collisions recorded (*p* = 0.002). In contrast, the proportion of collisions did not significantly differ between adults and children (*p* = 0.333) and between older adults and children (*p* = 0.087).

#### Use of prospective information for gap selection

**Fig. 3:**
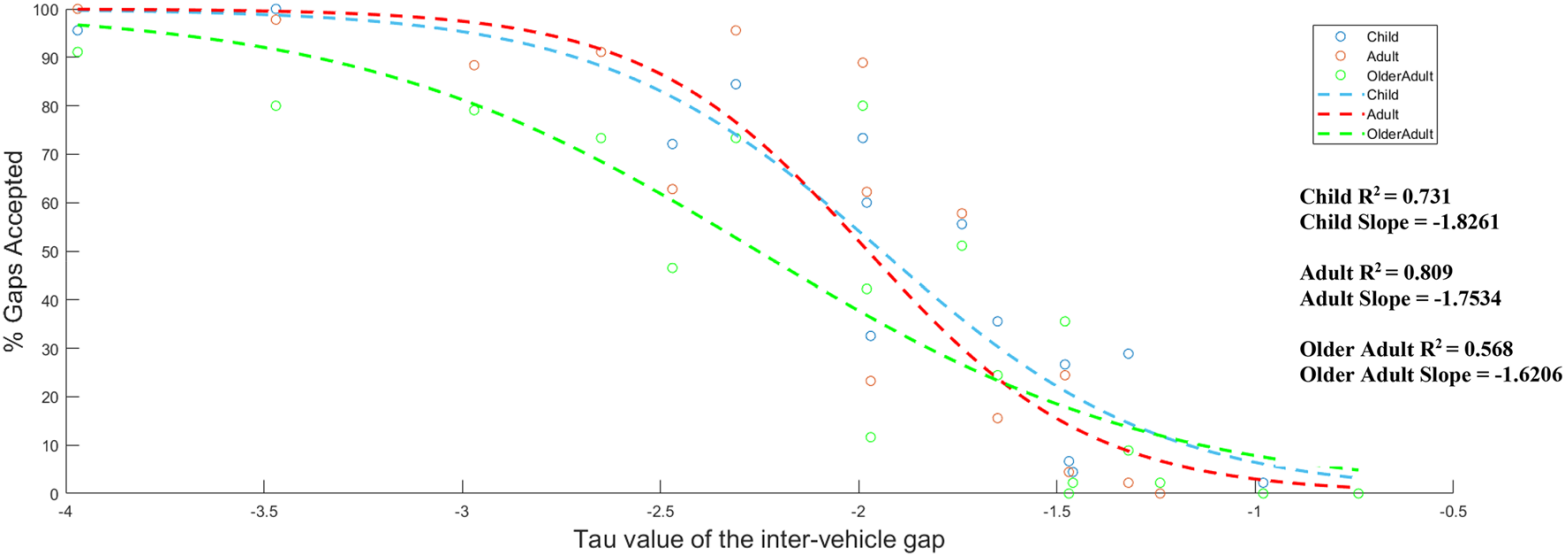
Figure showing the logistic functions (dashed lines) for the various tau values of the inter-vehicle gap when the gap first opens and the % average cross responses (circles) for children (blue), adults (red), and older adults (green).

To assess adherence to tau, a logistic (S-shaped) function was fitted between the tau of the inter-vehicle gap and the rate of gap acceptance data (see Fig. 3). The expected ‘S shape’ is revealed for children and adults where a high percentage of cross judgements are found for large tau values and a low percentage of cross-judgment for small tau values. The *r*^2^ values, calculated by the logistic regression, show that both children and adults adhere to a tau-based strategy (73.1% and 80.9% of the data, respectively). In contrast, the tau value of the inter-vehicle gap only explained 56.8% of the variance in the decision response data for older adults. This suggests that ageing influences the ability to pick up and use tau-based information to make decisions about the passability of inter-vehicle gaps.

### Action timing

#### Timing of entry

Once the decision to accept the gap has been selected, participants must tightly time their entry behind the lead vehicle to maximize the available time for crossing. To investigate whether age impacts on the ability to time one’s action to cross between the two vehicles, a one-way ANOVA was carried out. The results revealed that the mean timing of entry varied significantly by age group (*F*(2,44) = 4.740, *p* = 0.014, *η*^2^ = 0.18). Children recorded the tightest entry between the lead car passing and entering the inter-vehicle gap (*M* = 0.52 s, SD = 0.2), adults recorded the second-tightest entry, (*M* = 0.59 s, SD = 0.27), while older adults recorded the longest time before initiation of crossing (*M* = 0.93 s, SD = 0.59). A post hoc Tukey HSD test indicated that both child and adult pedestrians timed their entry significantly closer behind the lead vehicle compared to older pedestrians (*p* = 0.017 and *p* = 0.05, respectively). However, adults did not differ significantly from children (*p* = 0.889).

#### Variability of timing of entry

While older adults appear not to be attuning to the initial value of tau, when the inter-vehicle gap first opens, it is not clear whether longer initiation times are due to poorer adherence to prospective information or an age-related decline in timing skills. To assess the differences between age groups in their movement timing precision, the variability of timing of entry for each participant was also calculated (see Table 2). Adults were the least variable in their timing of entry (*M* = 0.31 s), older adults were the second-least variable (*M* = 0.39 s), and children were the most variable (*M* = 0.42 s); however, a one-way ANOVA revealed no significant main effect of variability of timing of entry for the three groups (*F*(2,44) = 1.119, *p* = 0.336, *η*^2^ = 0.01). These findings suggest that the age-related differences in mean timing of entry were intentional perception–action strategies rather than a result of age-related differences in the variability of motor control.

**Table 2:** Means (SDs) for each age group for action timing measures, including initiation time, timing variability, crossing time, and time to spare for adults, children, and older adults in the road-crossing task

Age group	Initiation time (s)	Timing variability (s)	Crossing time (s)	Time to spare (s)
Children	0.52 s (0.2)	0.42 (0.24)	3.62 (0.42)	2.01 (0.77)
Adults	0.59 (0.27)	0.31 (0.17)	3.8 (0.62)	2.28 (0.42)
Older Adults	0.93 (0.59)	0.42 (0.24)	3.82 (0.63)	1.77 (0.74)

### Action control

#### Road crossing duration

Given that average crossing speed and crossing duration are highly correlated across all groups (*r*^2^ = 0.769, *p* < 0.001), we opted to perform an ANOVA analysis with only the road-crossing duration. A one-way ANOVA showed no effect of age for mean road-crossing time (*F*(2,44) = 0.558, *p* = 0.576, *η*^2^ = 0.03). On average, children took 3.8 s to cross the road (SD = 0.62), adults took 3.62 s (SD = 0.42), and older adults took 3.82 s (SD = 0.63). This suggests no age differences in the ability to scale their walking speed to the spatio-temporal demands of the task imposed by speed and distance of the tail vehicle.

#### Duration of trial spent on a safe course of action

If older adults waited longer to initiate their crossing but had similar crossing times, one would expect that older adults had a smaller portion of the crossing event where their crossing afforded safe passage. This would mean that they would have more error to cancel out between the current state (current crossing speed affording collision) and ideal state (current crossing speed affording safe passage). It follows that if a participant’s current rate of gap closure between where they are currently and the opposite sidewalk (tauZ) is less than the current rate of gap closure between the tail vehicle and the participant (tauX), then the participant will be on collision course with the tail vehicle. A one-way ANOVA revealed that the average percentage of the trial in which the participant was on a safe course varied significantly by age group (*F*(2,44) = 7.621, *p* = 0.002, *η*^2^ = 0.27) indicating that age influenced the amount of time spent during crossing where their behaviour afforded safe passage. Adults recorded the highest percentage of the cross on a safe course of action (*M* = 92.7%, SD = 3.9), children recorded the second-highest percentage, (89.77%, SD = 6.9), and older adults recorded the lowest percentage of the cross where the rate of gap closure afforded safe passage (*M* = 79.6%, SD = 14.7). A post hoc Tukey HSD test indicated that older adults spent significantly less time on a safe course of action compared to both adults (*p* = 0.002) and children (*p* = 0.016). Adults did not differ significantly from children (*p* = 0.686) indicating that children and adults implemented a more effective perception–action coupling strategy that allows for safe crossing.

#### Magnitude of safety

**Fig. 4:**
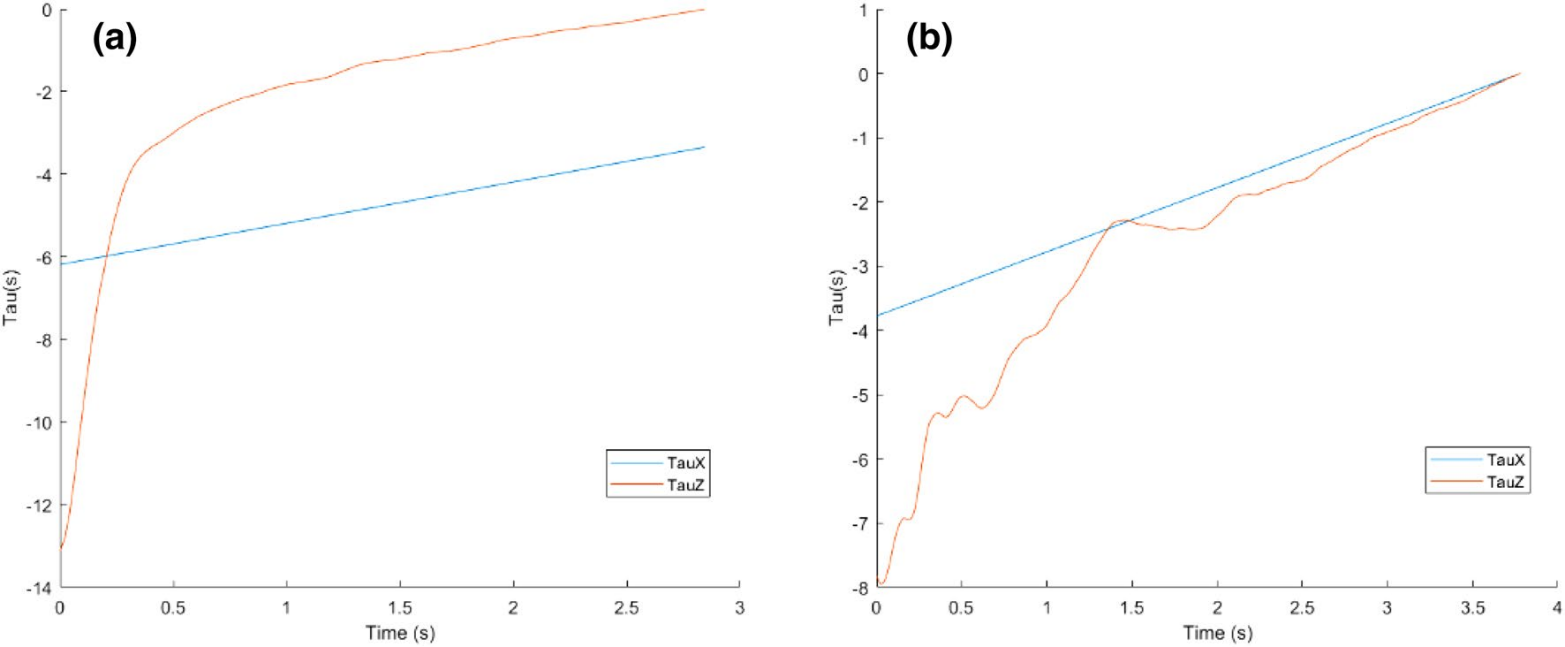
**a** Figure showing an adult’s successful cross represented by tauZ (action gap) closing (i.e. reaching zero) above tauX (information gap). **b** Figure showing an older adult’s unsuccessful cross represented by tauX (information gap) closing (i.e. reaching zero) above tauZ (action gap)

Once a participant uses tau information to reach a state in which they are no longer on a collision course (i.e. the tau differential is positive), we assessed if there were group differences in the ability to *maintain* safe crossing. If participants were using tau information to guide their crossing actions on-line, one would expect the participant to modulate their crossing speed in such a way to ensure they closed the action gap (gap between curbs) faster than the information gap (gap between vehicles) and then keep a large aperture between these gaps to guarantee safe passage. One strategy would be to accelerate early after an action is initiated to obtain a positive tau difference (and a sufficient crossing speed) and then maintain that crossing speed for the remainder of the cross. In contrast, participants who were less skilled in using such information would struggle to maintain a large aperture between the two taus and continually alternate between a positive and negative tau differential. For instance, Fig. 4a shows an example of a trial involving an adult who recorded zero collisions and a high average magnitude of safety (*M* = 3.27). The participant appeared to get a positive tau differential early during the crossing sequence, then control their crossing speed to maintain a large temporal safety margin. In contrast, Fig. 4b shows an example of a trial involving an older adult with a higher percentage of collisions from accepted crosses (50%) and a lower average temporal safety margin (*M* = 0.84). In this example, the participant struggled to maintain a walking speed that was sufficient and as a result the information gap (represented by tau *X*) closed before they could close the action gap (represented by tau *Z*). Indeed, analysis of the mean area under curve using the Trapezoidal Integration Method indicated that children (*M* = 2.28, SD = 0.52) and adults (*M* = 2.28, SD = 0.61) maintained a larger temporal margin of safety compared to older adults (*M* = 1.85, SD = 0.57). These findings are in keeping with the higher levels of successful crosses found in both these groups. However, that being said, the main effect of group failed to reach significance (*F*(2,44) = 2.895, *p* = 0.066, *η*^2^ = 0.12).

#### Time to spare

To investigate if age affected the amount of time remaining until the tail car arrived at the intersection; mean time to spare (seconds) was also calculated. A larger value of time to spare would reflect a larger aperture between the information gap (tau X) and the action cap (tau Z), at the point the participant reached the opposite sidewalk. A one-way ANOVA revealed no significant differences for mean time to spare across all three age groups (*F*(2,44) = 2.187, *p* = 0.125, *η*^2^ = 0.01). On average, children had 2.01 s (0.77) before the tail vehicle closed the gap, adults had the most time to spare at 2.28 s (0.42) and older adults had the least time to spare at 1.77 s (0.74).

## Discussion

### Summary of findings

The aim of the present experiment was to understand how ageing impacts on the use of higher-order optical information to support both action-based decisions and the continuous regulation of movement when crossing a road in a dynamic traffic environment. Lee (1976) presented a conceptual framework for understanding how information directly available to the observer (namely tau) can be used to fit actions into spatio-temporal windows. In the present task, tau theory was found to support both decisions about the ‘cross-ability’ of various inter-vehicle gaps and the control of actions to ensure the gap between curbs was closed before the vehicle reached the participant. However, older adults showed greater difficulty tuning into this information before the inter-vehicle gap opened, an observation that was reflected in the initial tau value predicting only 56.8% of the variance found in gap acceptance, compared to 73.1% in children and 80.9% in adults. This lower adherence to prospective information extended beyond action selection and directly influenced initiation times and how self-movement was controlled with respect to the approaching car. The present experiment furthers our understanding as to why different age groups performed differently in road-crossing tasks where the participant is free to regulate their actions based on the information they pick up through their movement.

### Unifying action selection and control through perceptual information

In the present study, tau theory was found to provide an elegant, parsimonious model to explain how action selection and action timing and control can be regarded as the one and the same dynamic process supported by adherence to a single source of information (Cisek, 2007; Stoffregen, 2000). For action selection, the initial value of the invariant tau specifies how the gap between the pedestrian and the vehicle is closing over time at the instant the inter-vehicle gap opens. For action timing and control, modelling the situation as the closure of two gaps, one between the pedestrian and the opposite curb and one between the pedestrian and the approaching vehicle, provides a parsimonious solution as to how pedestrians can cross the inter-vehicle gap (at the current closure rate), by tuning into the relative difference between these current gap closures. Of course, being able to demonstrate that tau can theoretically and mathematically explain the situation does not necessarily mean that it is an informational variable that we use or are attuned to when making such judgments (Watson et al., 2011). Indeed, researchers have proposed other methods of determining when objects will arrive at the point of observation, particularly when they are often out of view. For example, Tresilian (1995) proposed that an observer could make an initial estimation of TTA before the object went out of view (in the case of the present experiment looking at the other lane) and then use a cognitive clocking process to count down the time until the disappeared object reached the observer. However, due to the symmetrical nature of both lanes in the present experiment, the observer would only need to accurately determine the time to closure of one inter-vehicle gap once the lead vehicle had passed the participant removing the need for any clocking mechanism. This is because the variables available in the looming optic array, and accessible to the observer, correspond with the virtual vehicle’s motions towards the point of observation (Bootsma & Oudejans, 1993). Since the time to closure of the inter-vehicle gap remains the same in the present virtual traffic environment, regardless of which lane the participant is attuning to, no cognitive model of the out-of-view vehicle’s motion is required.

Despite the uncertainty over how exactly the TTA judgements were being made, the fact that an ‘s-shaped’ curve was revealed for children and adults when ‘% of gaps judged as safe to cross’ was plotted as a function of the approaching vehicle’s initial tau value, suggests that prospective judgments were indeed being made by these age groups when selecting an inter-vehicle gap to cross through. Furthermore, the fact that children and adults initiated their actions sooner and modulated their crossing behaviours in a manner consistent with what would be expected according to tau theory (i.e. avoiding being on a collision course earlier and maintaining a large aperture between the two gaps) suggests that actions were being prospectively controlled.

Indeed, this road-crossing task emphasises the challenges faced by elderly populations when perceiving and acting on dynamic affordances involving whole body movement. When selecting gaps, we observed clear age-related decline in both gap thresholds and sensitivity. Despite crossing the least number of times, older adults recorded the highest number of collisions and were less discriminating than children and adults in what temporal gap afforded safe passage. Although the present task allowed a tau-based strategy by designing a scenario that involved the closure of two motion gaps (e.g. Lee, 1998; Stafford et al., 2019), older adults adhered the least to this optical parameter when judging ‘crossability’. We found older adult’s lower adherence to initial tau-based information when selecting an appropriate inter-vehicle gap directly influenced how that gap was subsequently acted upon. Children and adults showed they could prospectively control their actions by tightly initiating their crosses as soon as the inter-vehicle gap opened. In contrast, older adults waited longer on the curb and exhibited less skill when regulating their crossing behaviour with respect to the approach of the oncoming vehicle.

### Children’s action decision-making

What might underlie children’s ability to use higher-order variables to act ahead of time in the present virtual traffic environment? Research investigating developmental changes in interceptive actions suggests that the process of gradually converging onto the use of the information specifying the TTA of an approaching object begins during infancy as a method of preventing damage to the sensitive cornea of the eyes (Caljouw, Van der Kamp, & Savelsbergh, 2004; van der Meer, van der Weel, & Lee, 1994). It seems plausible then, that the ability to judge when they are on a collision course with an approaching object begins to develop from a young age as an adaptive method of self-preservation from unintended injury. Indeed, at a young age, children have been shown to use this temporal information for both the timing of movement initiation and online guidance of movement (Kayed & van der Meer, 2009; van Hof, van der Kamp, & Savelsbergh, 2008). However, the time frame of when children can finely tune gap choices and crossing behaviour is still uncertain. For instance, when crossing a single lane of traffic in a cycling simulator, both 10- and 12-year-old are less adept than adults in timing their movement compared to adults (Plumert et al., 2004). In contrast, when crossing through a single lane of traffic on foot, 12-year-old appear to have learned to adjust their gap selection to more closely match their action capabilities. (O’Neal et al., 2018). O’Neal and colleagues argue 12-year-old were able to outperform their younger counterparts when crossing roads on foot because at that age children have developed enough movement skill to become more “aware” of their perceptual-motor limitations. However, it was noted that this ability had still not reached adult-like levels suggesting the coupling of perception of action possibilities and their subsequent actualization continues to develop into early adolescence.

The tight timing of entry of the child group found in the present study contradicts previous research showing significant timing differences between children aged 10 and 12 years and adults. One possible reason for this discrepancy is that the children in the present study were required to traverse two lanes of traffic and thus travel a greater distance on foot, placing a greater demand on their ability to calibrate the gap size to their action capabilities. Indeed, previous research has shown that when higher task demands are experimentally implemented on an actor, the strength of the perceptual-motor regulation is increased (van Andel, Cole, & Pepping, 2018). In traffic environments, this would ultimately result in an increase in prospective control of actions. For instance, Lobjois and Cavallo (2009) show pedestrians can compensate for limitations in their motor systems when physically crossing roads by timing the entry behind the lead vehicle of the gap into the roadway more tightly to maximize the time available to reach the opposite sidewalk.

Another possible explanation for this discrepancy is the differences in technology used to investigate action decision-making. In contrast to previous research which used CAVE (Computer Assisted Virtual Environment) systems consisting of three right-angled screens forming a three-walled room, the present experiment adopted HMD technology. Recently, Mallaro, Rahimian, O’Neal, Plumert, and Kearney (2017) found that in a virtual road-crossing task, participants wearing an HMD timed their entry into the inter-vehicle gap more precisely and tended to cross more quickly compared to the CAVE group. The authors proposed that this finding may be due to the fact that HMDs offer greater pixel density per visual angle thus providing superior cues for TTA judgements (such as the magnitude of a vehicle’s optical solid angle and its current rate of change). Indeed, such an interpretation would support why children in the present study had greater prospective timing of their movements. Since this age group has been shown to have reduced sensitivity to visual looming (Wann et al., 2011), they may take greater benefit from technology that makes TTA information more salient.

### Older adults’ action decision-making

In contrast to the results found for children, the current experiment proved challenging for older adults. In general, coupling perception and action under tight temporal constraints, such as crossing the road, is particularly demanding for older adults due to age-related declines in the perceptual-motor system. Perceptually, visual contrast deteriorates (Pitts, 1982), sensitivity of peripheral vision declines (Jaffe, Alvarado, & Juster, 1986), and discrimination between rates of looming depreciates (Poulter & Wann, 2013). Furthermore, ageing is associated with physical decline as muscles weaken resulting in a decline of maximum achievable movement speed, isometric and dynamic strength (Larsson, Grimby, & Karlsson, 1979). These declines in the perceptual-motor system mean that older adults need to cope with the changing relationship between perceptual and motor function.

In regard to gap selection, older adults were less discriminating than adults and children indicating that they sometimes took very small temporal gaps and sometimes missed large temporal gaps. Such a finding is reinforced by the older adult’s reduced sensitivity to tau information which specifies TTA irrespective of the approaching vehicle’s initial distance or speed. As a result, it is possible that older adults often had distorted affordance judgements resulting in sometimes overestimating or underestimating the temporal window for action. The finding that one age group is less sensitive to a particular perceptual variable is consistent with the ‘direct’ approach to perception and learning in which expertise is reflected in the ability to rely on higher-order variables available in the optic flow field (Jacobs & Michaels, 2007). Recent research has suggested that the perceptual variables (both specifying and non-specifying) attended to in traffic environments are malleable to training (Stafford & Rodger, 2020). Therefore, more research investigating protocols for training older adults’ ability to tune into TTA information is of particular importance.

Furthermore, the findings in action timing suggest that older adults may compensate for this reduced sensitivity by delaying crossing behind the lead vehicle in an attempt to wait for the TTA information from the tail vehicle to become more obvious. For instance, if they wait longer to initiate crossing, they observe the vehicle for longer, which in turn might improve their ability to detect how fast the car is approaching (e.g. extracting vehicle velocity information from the change in optical size over time i.e. the expansion rate (Poulter & Wann, 2013)). As optical tau determines the TTA of an approaching object as the ratio of the instantaneous optical size of an object relative to its rate of expansion on the retina, any decrement in sensitivity to the rate of expansion would negatively impact upon the ability to pick up tau information. Crucially, however, time spent judging the affordance of a gap takes time away from actualising the affordance by executing the action (Fajen, 2013; Plumert & Kearney, 2014). It appears that in the present task, older adults were faced with a trade-off between timing their actions early to achieve the largest temporal window to complete the act of crossing and waiting longer to better cope with their limited sensitivity to the vehicle’s rate of looming. Our findings suggest that older adults prioritise the latter, delaying the initiation of crossing to provide an indirect way of compensating for limited ability to detect higher-order information (explained by the lower adherence to initial tau to explain road-crossing decisions). As a vehicle gets closer to the pedestrian, the visual information required to specify its TTA becomes more salient and easier to detect (e.g. Wann, Poulter, & Purcell, 2011) and use to regulate action. Nevertheless, an older adult must continuously balance the desire to delay crossing initiation with the fact that executing the act of crossing will take time. The present findings showed that older adults spent a smaller portion of the crossing event on a safe crossing course with respect to the approaching vehicle (i.e. maintaining a safe temporal margin). Movement initiation should therefore not only rely on features of the vehicle’s motion, but also on the to-be performed action. Indeed, an important factor when crossing the road is the ability to scale decisions and actions in relation to the information arising from the oncoming vehicle, a process known as perceptual-motor calibration (Bingham & Pagano, 1998; Warren, 1984; Withagen & Michaels, 2007).

### Limitations and conclusion

Despite the insights that can be drawn from this study, there were some limitations and further questions that need addressed. First, the present study only looked at children from a narrow age range in middle childhood (10–12 years). Indeed, recent research suggests that childhood is associated with ongoing developmental refinement in how to perceive and act on dynamic affordances when crossing roads (Chihak et al., 2014; Plumert & Kearney, 2014; Plumert, Kearney, Cremer, Recker, & Strutt, 2011). Interestingly, around the age of 12, children appear to begin to adjust their gap choices to closely match their action capabilities in static (Franchak, 2019) and dynamic (O’Neal et al., 2018) environments. O’Neal et al. (2018) notes that for the task of physically walking across the road, children younger than 10 years old have not yet gained sufficient movement skills for accurate perceptuo-motor calibration. This suggests that a child’s movement competence limits the ability to map the perceptual information arising from the oncoming cars to the actor’s action capabilities.

Second, although our task involved crossing two lanes of traffic, the vehicles were not accelerating and were synchronized to arrive at the same time to create an aligned gap (far gap opens with near gap). As a result, participants only needed to attend to the tail vehicle in the far gap once the gap opened to ensure safe passage. Quite possibly, even 12-year-old might have had more difficulty than adults integrating TTA information in more complex situations, such as rolling traffic gaps (far gap opening after near gap). Grechkin et al. (2013) found that children preferred rolling gaps as they appeared to acknowledge that despite being the more complex crossing strategy, rolling gaps have potential for extending the total possible crossing time. Future research should investigate if older adults exhibit the same preference, timing their actions earlier when the gaps are not aligned to maximise the temporal window for action and counteract the delayed movement initiation found in the present study.

Finally, while head-mounted displays present many advantages for this line of research including their widespread availability, low cost, immersive qualities, and ability to present an egocentric viewpoint, it does present some corresponding issues. Notably, the fact that HMDs typically have a more restricted field of view compared to CAVE virtual reality systems (e.g. 100 degrees of the Oculus Rift compared to 170 degrees in the VicCube CAVE) may have impacted the scanning behaviours of participants, who would require more pronounced head movements when monitoring the bi-directional traffic compared to CAVE systems. Additionally, the encumbrance of a head-mounted display and the necessity of managing a cable may have altered the movement of participants, particularly for older adults who place greater emphasis on maintaining balance while walking in virtual environments (Woollacott and Pei-Fang, 1997). While no participants were unable to complete the experiment due to motion sickness, future work should explore whether CAVEs or HMDs are more likely to induce motion sickness in both child and older adult populations. This will help inform what methodological tool is most appropriate for designing training protocols.

In conclusion, the results of this study highlight the importance of considering how the characteristics of the individual and the properties of the environment jointly influence the ability to couple perception and action in the context of crossing roads. The findings show that the ability to use higher-order information to link decisions and actions is sensitive to age-related decline and as a result, impacts an older adult’s ability to select actions ahead of time and tightly time their entry into the roadway. To compensate for the reduced sensitivity to prospective information, older adults appear to wait longer, but with detrimental consequences in terms of using the time available to safely cross the road and avoid colliding with the oncoming vehicle. Age-related differences in using perceptual information which guides when and how to act, in conjunction with the relationship between perception and action coupling, should thus be considered in the contexts where errors in action-based decisions are likely to occur.

## Data accessibility

Data supporting the findings of this study are available from the corresponding author on request.
